# Association of Serum Calprotectin Concentrations with Mortality in Critically Ill and Septic Patients

**DOI:** 10.3390/diagnostics10110990

**Published:** 2020-11-23

**Authors:** Theresa H. Wirtz, Lukas Buendgens, Ralf Weiskirchen, Sven H. Loosen, Nina Haehnsen, Tobias Puengel, Samira Abu Jhaisha, Jonathan F. Brozat, Philipp Hohlstein, Ger Koek, Albrecht Eisert, Raphael Mohr, Christoph Roderburg, Tom Luedde, Christian Trautwein, Frank Tacke, Alexander Koch

**Affiliations:** 1 Department of Medicine III, University Hospital RWTH Aachen, Pauwelsstraße 30, 52074 Aachen, Germany; thwirtz@ukaachen.de (T.H.W.); lbuendgens@ukaachen.de (L.B.); nina.haehnsen@rwth-aachen.de (N.H.); sabujhaisha@ukaachen.de (S.A.J.); jbrozat@ukaachen.de (J.F.B.); phohlstein@ukaachen.de (P.H.); ctrautwein@ukaachen.de (C.T.); 2 Institute of Molecular Pathobiochemistry, Experimental Gene Therapy and Clinical Chemistry, University Hospital RWTH Aachen, Pauwelsstraße 30, 52074 Aachen, Germany; rweiskirchen@ukaachen.de; 3 Clinic for Gastroenterology, Hepatology and Infectious Diseases, University Hospital Düsseldorf, Medical Faculty of Heinrich Heine University Düsseldorf, Moorenstraße 5, 40225 Düsseldorf, Germany; sven.loosen@med.uni-duesseldorf.de (S.H.L.); tom.luedde@med.uni-duesseldorf.de (T.L.); 4 Department of Hepatology and Gastroenterology, Charité University Medicine Berlin, Augustenburger Platz 1, 13353 Berlin, Germany; tobias.puengel@charite.de (T.P.); raphael.mohr@charite.de (R.M.); christoph.roderburg@charite.de (C.R.); frank.tacke@charite.de (F.T.); 5 Section of Gastroenterology and Hepatology, Department of Internal Medicine, Maastricht University Medical Centre (MUMC), 6229 HX Maastricht, The Netherlands; gh.koek@mumc.nl; 6 Hospital Pharmacy, RWTH-University Hospital Aachen, 52074 Aachen, Germany; aeisert@ukaachen.de; 7 Institute of Clinical Pharmacology, RWTH-University Hospital Aachen, 52074 Aachen, Germany

**Keywords:** intensive care unit, biomarker, sepsis, prognosis, organ failure

## Abstract

**Background:** Calprotectin is present in the cytosol of neutrophil granulocytes and released upon activation. Fecal calprotectin is applied in the clinical management of inflammatory bowel disease whereas serum calprotectin has been discussed as a biomarker in inflammatory disorders. However, its long-term prognostic relevance in critical illness remains unclear. Our aim was to investigate serum calprotectin concentrations as a prognostic biomarker in critically ill and septic patients. **Methods:** Serum calprotectin concentrations were analyzed in 165 critically ill patients (108 with sepsis, 57 without sepsis) included in our observational study. Patients were enrolled upon admission to the medical intensive care unit (ICU) of the RWTH Aachen University Hospital. Calprotectin concentrations were compared to 24 healthy controls and correlated with clinical parameters, therapeutic interventions, and survival. **Results:** Serum calprotectin concentrations were significantly increased in ICU patients as well as in septic patients compared to respective controls (*p* < 0.001 for ICU patients and *p* = 0.001 for septic patients). Lower calprotectin concentrations were measured in patients with comorbidities i.e., coronary artery disease. Calprotectin concentrations strongly correlated with the C-reactive protein (*p* < 0.001) and were closely associated to parameters of mechanical ventilation (i.a. inspiratory oxygen fraction, FiO_2_; *p* < 0.001). The overall survival was significantly impaired in septic patients with high baseline calprotectin concentrations (*p* = 0.036). However, patients with increasing calprotectin serum concentrations within the first week of ICU admission showed an improved overall survival (*p* = 0.009). **Conclusions:** In summary, serum calprotectin concentrations are significantly increased in critically ill patients with sepsis. High calprotectin concentrations at ICU admission predict long-term mortality risk, whereas increasing calprotectin concentrations are associated with a favorable long-term outcome.

## 1. Introduction

Calprotectin represents a heterodimer of the mammalian proteins S100A8 and S100A9. It accounts for 60% of the soluble protein in the cytosol of neutrophil granulocytes and is released by neutrophils upon their activation and turnover [1,2]. Fecal calprotectin is an established marker for neutrophil mediated inflammation in the gut mucosa as it correlates with the numbers of infiltrating neutrophils and the severity of intestinal inflammation [3,4]. Calprotectin was furthermore characterized as a serum-based biomarker in several inflammatory diseases, i.e., juvenile rheumatoid arthritis [5] and systemic lupus erythematosus [6]. Since one of the main sources for S100A8/S100A9 secretion is infection-induced inflammation, calprotectin concentrations in serum were found to be elevated during bacterial infections [7,8,9,10] and moreover indicated complicated courses of disease [11]. Importantly, both S100 proteins were identified to participate in innate immunity in a proinflammatory manner as they mediate inflammatory responses by triggering cytokine release and neutrophil recruitment—both essential mechanisms in inflammatory responses and immune defense [12,13].

Bacterial infections may cause life-threatening conditions referred to as sepsis or septic shock. Here, a dysregulated immune response progresses to an organ dysfunction of varying degree, representing a substantial cause of death among critically ill patients [14]. An evidence-based risk evaluation of the patient’s individual clinical characteristics could increase the prognosis substantially [15]. Moreover, during the course of sepsis, clinical markers are needed to improve diagnosis and risk-adaptive treatment in critically ill patients. Besides clinical assessment and scores to evaluate organ failure [16], biomarkers may indicate patients at risk for unfavorable courses as well as impaired short- and long-term survival. 

Due to its role in bacterial induced inflammation, we hypothesized that calprotectin might play a relevant role in the course of critically ill patients with and without sepsis. As it is already known that calprotectin predicts short-term mortality in septic patients [17], we here investigate the association between circulating calprotectin and severity of critical illness as well as sepsis in a long-term perspective. Moreover, we ask whether calprotectin might function as a long-term prognostic biomarker in critically ill patients treated on a medical intensive care unit (ICU).

## 2. Material and Methods

### 2.1. Study Design and Patients’ Characteristics

This observational cohort study was performed to investigate the role of circulating calprotectin in critically ill patients treated on a medical ICU. A total of *n* = 165 patients who were admitted to the Department of Gastroenterology, Digestive Diseases, and Intensive Care medicine of the RWTH Aachen University Hospital between 2006 and 2012 were included in the study. Written informed consent was obtained from the patient, her/his spouse or the appointed legal guardian. As patients’ recruitment took place on a medical intensive care unit for adults, patients with an age below 18 years could not be included in the study. Moreover, patients with an expected short-term ICU stay of less than 72 h, e.g., due to post-operative observation, were excluded. As a control population, *n* = 24 healthy blood donors without severe acute or chronic disease and who are medically examined on a regular basis were included. The Third International Consensus Definition for sepsis was used to retrospectively discriminate sepsis and non-septic patients [14]. The study protocol was approved by the local ethics committee (ethics committee of the University Hospital RWTH Aachen, RWTH Aachen University, Aachen, Germany, reference number EK 150/06, 2 November 2006) and performed according to the ethical standards laid down in the 1964 Declaration of Helsinki.

### 2.2. Calprotectin Measurements

After admission to the ICU, blood samples were collected at day 1 and day 7. Samples were centrifuged at 4 °C for 10 min and serum aliquots of 1 mL were frozen immediately at −80 °C until use. Calprotectin serum concentrations were measured using a commercially available ELISA according to the manufacturer’s instructions (PhiCal Calprotectin ELISA Kit, Immundiagnostik AG, Bensheim, Germany). Calprotectin measurements were performed fully blinded to any clinical or other laboratory data of the patients or controls.

### 2.3. Statistical Analysis

Data are given as median and range due to the skewed distribution of most of the parameters. Box plot graphics are used to illustrate differences between subgroups. All values have been included for statistical analyses. Differences between two groups were assessed by Mann–Whitney U test. Correlations between variables were analyzed by linear regression analyses. To investigate prognostic value of the variables, univariate analysis using the Cox regression model was performed. Bootstrapping was added as an internal validation for the predictive value of calprotectin. Variables correlating with overall survival with a *p*-value of <0.200 in univariate testing were included in a multivariate Cox regression analysis using a stepwise backward procedure with calprotectin as the dependent variable to find independent predictors of patients’ outcome. In order to illustrate differences in survival, Kaplan–Meier curves were plotted. The log-rank test was used to test the level of significance. The ideal cut-off value for the identification of patients with an impaired OS was calculated by fitting Cox proportional hazard models to the dichotomized survival status as well as survival time and defines the optimal cut-off as the point with the most significant split in the log-rank test [18]. All statistical analyses were performed with SPSS Version 23 (SPSS, Chicago, IL, USA).

## 3. Results

### 3.1. Patients’ Characteristics

In our analysis, *n* = 165 patients were included who were admitted to our medical ICU due to critical illness. The median age of the study cohort was 64 years with a range from 18 to 90 years. In total, 59.4% of ICU patients were male and 42.4% were female. In addition, 31.6% of included patients had previously been diagnosed with diabetes mellitus type 2; 10% suffered from malignant disease at the time point of ICU admission. The underlying cause of ICU admission was distributed as follows: 65.5% sepsis, 13.9% cardiopulmonary disease, 5.5% acute pancreatitis, 4.2% decompensated liver cirrhosis, 2.4% gastrointestinal bleeding, 1.2% acute liver failure, 7.3% others. The main site of infection in patients admitted due to sepsis was pulmonary (55.6%). Further detailed patient characteristics are summarized in Table 1.

### 3.2. Calprotectin Serum Concentrations Are Increased in Critically Ill and Septic Patients 

**Figure 1:**
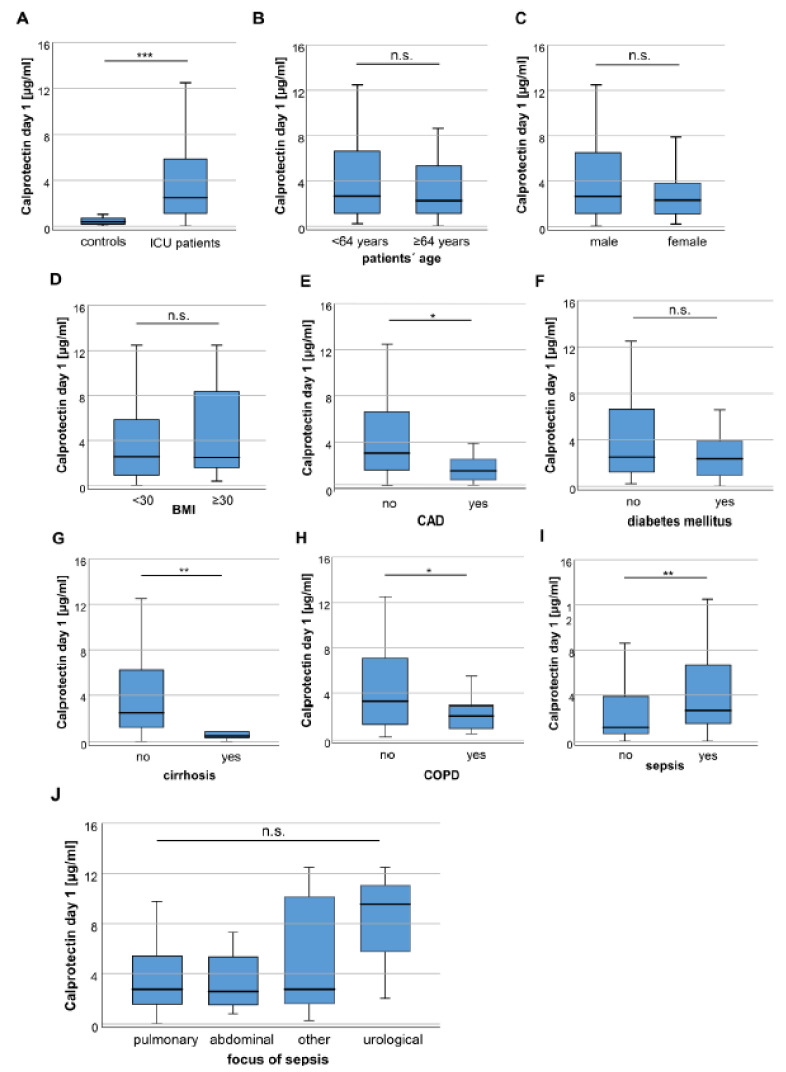
Calprotectin serum concentrations are significantly higher in critically ill and sepsis patients. (**A**) Critically ill patients at admission to the ICU have significantly lower calprotectin serum concentrations compared to healthy controls. (**B**) Calprotectin serum concentrations did neither differ between patients younger or older than 64 years nor between male and female patients (**C**) or patients with a body mass index (BMI) below or above 30 (**D**). (**E**) Patients with coronary artery disease (CAD) but not diabetes mellitus (**F**) showed significantly lower calprotectin concentrations. (**G**) Patients with liver cirrhosis or chronic obstructive pulmonary disease (COPD) (**H**) also had significantly lower calprotectin serum concentrations at day 1 of ICU admission. (**I**) Calprotectin concentrations are significantly increased in patients admitted due to sepsis compared to patients without. (**J**) Different sites of infections in patients with sepsis did not result in significantly different serum concentrations of calprotectin. * *p* < 0.05; ** *p* < 0.005; *** *p* < 0.001, n.s.: not significant.

We compared serum calprotectin concentrations between patients at the time-point of ICU admission and healthy control samples. In these analyses, we observed significantly higher calprotectin serum concentrations in the patients’ group (3.97 µg/mL vs. 2.48 µg/mL in controls, *p* < 0.001; Figure 1A). Subsequently, we aimed at evaluating whether calprotectin serum concentrations are altered regarding different demographic subgroups. However, we did not observe a significant alteration of calprotectin serum concentrations between patients younger or older than 64 years (median of study cohort, Figure 1B), male and female patients (Figure 1C), as well as between patients with a body mass index (BMI) below or above 30 kg/m^2^ (Figure 1D). In a next step, we analyzed if chronic comorbidities of ICU patients influence circulating calprotectin concentrations. Patients with coronary artery disease (CAD) showed significantly lower concentrations of calprotectin compared to patients without CAD (*p* = 0.007, Figure 1E). In contrast, calprotectin serum concentrations were comparable between patients with and without diabetes mellitus (*p* = 0.498, Figure 1F). We observed a significant difference of calprotectin serum concentrations between patients with or without liver cirrhosis (*p* = 0.001, Figure 1G) and patients suffering from chronic obstructive pulmonary disease (COPD) compared to patients without COPD (*p* = 0.047, Figure 1H). Interestingly, patients with sepsis had significantly higher calprotectin serum concentrations (*p* = 0.001, Figure 1I). Moreover, calprotectin concentrations were independent of the site of infection since patients with different sepsis focus had comparable calprotectin serum concentrations (Figure 1J).

### 3.3. Calprotectin Serum Concentrations in ICU Patients Positively Correlate with Markers of Systemic Inflammation and Parameters of Mechanical Ventilation

To further investigate potential drivers of increased calprotectin concentrations in ICU patients, we performed extensive linear regression analyses between calprotectin and clinical parameters of critically ill and septic patients including markers of organ dysfunction. Here, calprotectin concentrations did not correlate with duration of ICU or hospital stay neither in all critically ill patients included in the analysis nor in septic patients. Correlation of calprotectin serum concentrations with markers of systemic inflammation at ICU admission revealed a significant correlation between calprotectin and CRP serum concentrations both in critically ill (regression coefficient, r: 17.63 with 95% confidence interval, CI, 10.64–24.62; *p* < 0.001) as well as septic patients (r: 17.67, 95% CI 7.01–28.33; *p* = 0.001). However, no correlation was found for calprotectin concentrations and leukocytes, procalcitonin or interleukin 6 neither in critically ill nor septic patients. Regarding laboratory markers of organ function, we dissected a correlation of calprotectin concentrations with duration of renal replacement therapy (RRT, r: 186.97, 95% CI 59.41–314.53; *p* = 0.004) and urea (r: 12.74, 95% CI 1.22–24.26; *p* = 0.030) in critically ill patients but not septic patients, even if there was a trend regarding the correlation of RRT duration with calprotectin concentrations in this subgroup (r: 130.45, 95% CI 10.99–272.0; *p* = 0.070). Creatinine concentrations or the glomerular filtration rate (GFR) did not show a significant correlation. Additionally, we did not observe a correlation between calprotectin concentrations and markers of an impaired liver or cardiac function (AST, bilirubin; NT-proBNP; Table 2).

Calprotectin concentrations did not significantly correlate with scores of disease severity such as acute physiology and chronic health evaluation (APACHE II) score, sequential organ failure assessment (SOFA) score and simplified acute physiology (SAPS II) score. Nevertheless, calprotectin concentrations were found to be associated with severity of respiratory failure, as investigation of parameters of mechanical ventilation revealed: Here, the fraction of inspired oxygen (FiO_2_), the maximum airway pressure (P_max_), and the level of positive end-expiratory pressure (PEEP) at first day of admission significantly correlated with calprotectin concentrations both in critically ill and septic patients (Table 2). However, calprotectin concentrations did not differ between patients with and without dependence of mechanical ventilation (data not shown); also, duration of mechanical ventilation was not associated with higher calprotectin concentrations (Table 2).

### 3.4. Baseline Calprotectin Serum Soncentrations Predict Long-Term Survival in Septic Patients

**Figure 2:**
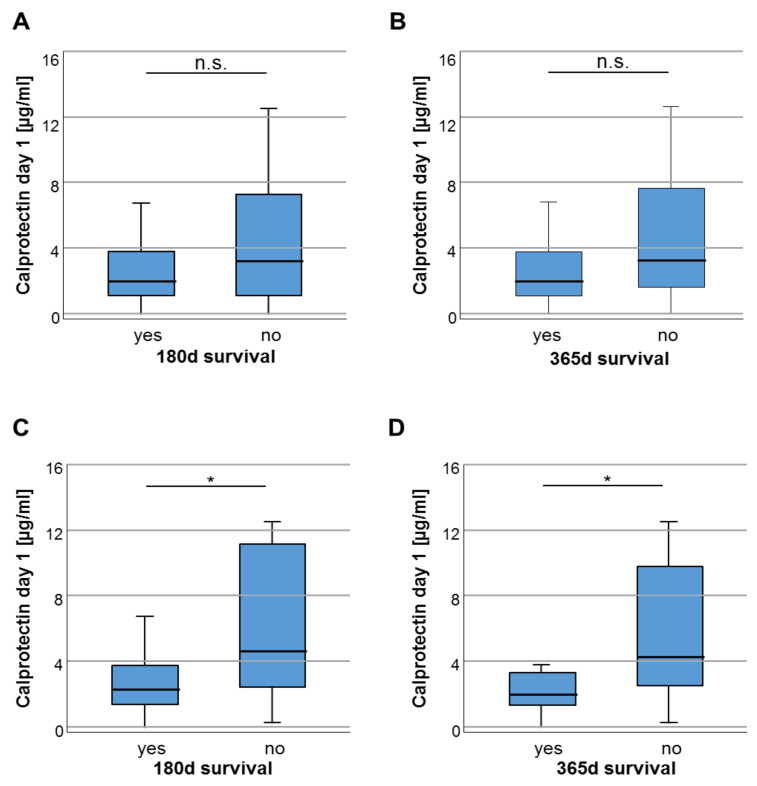
High baseline calprotectin concentrations predict poor 180 and 365 days outcome in septic patients. The subfigures (**A**,**B**) include the whole cohort of critically ill patients whereas subfigures (**C**,**D**) are based on subcohort data of patients admitted to the ICU due to sepsis. (**A**) Critically ill patients who died 180 days or 365 days after ICU admission show a trend towards higher calprotectin serum concentrations (*p* = 0.104 for 180 d; A; *p* = 0.076 for 365 d, B). (**C**,**D**) Septic patients who deceased during the first 180 or 365 days following ICU admission show significantly higher calprotectin concentrations compared to survivors (*p* = 0.019 for 180 d; E; *p* = 0.013 for 365 d, F). * *p* < 0.05; n.s.: not significant.

As it is known that calprotectin serum concentrations predict short-term mortality in critically ill patients [17], we next hypothesized that the increased concentrations of circulating calprotectin in critically ill and septic patients could indicate the individual patient’s long-term outcome. First, the 180 and 365 days mortality after ICU admission was investigated. We compared serum calprotectin concentrations in critically ill patients who survived the first 180 or 365 days respectively after ICU admission to patients who deceased during this time period. Here, we revealed a trend towards higher calprotectin concentrations in patients who did not survive the respective time-point (*p* = 0.104, Figure 2A and *p* = 0.076, Figure 2B), even if these results did not reach statistical significance. Next, we investigated the impact of calprotectin serum concentrations on the long-term mortality in patients fulfilling sepsis criteria. In this subgroup, we observed significantly higher calprotectin concentrations in sepsis non-survivors after 180 and 365 days (*p* = 0.019, Figure 2C; *p* = 0.013, Figure 2D).

### 3.5. Increasing Calprotectin Serum Concentrations during the Course of Critical Illness Indicate an Improved Overall Survival 

**Figure 3:**
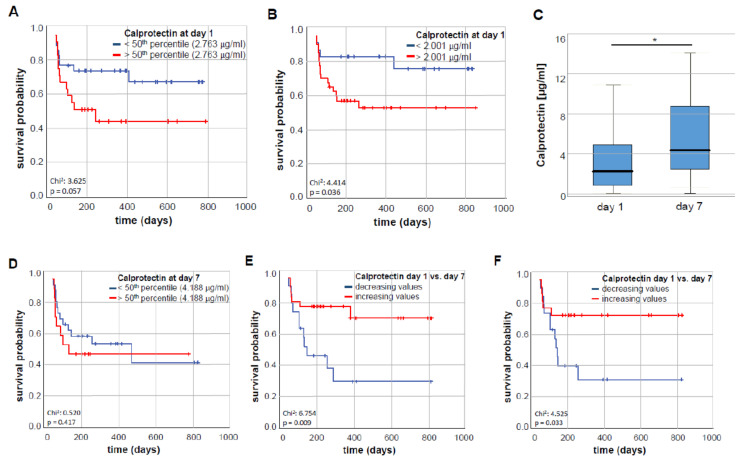
Calprotectin serum concentrations during the course of critical illness. (**A**) Using the median calprotectin serum level (2.763 µg/mL) as cut-off value, Kaplan–Meier curve analysis reveals a trend towards an impaired overall survival (OS) in septic patients with high calprotectin serum concentrations at ICU admission (*p* = 0.057). (**B**) When the ideal cut-off value was applied, septic patients with a baseline calprotectin serum level above 2.001 µg/mL have a significantly impaired OS compared to patient with calprotectin concentrations below the cut-off value (*p* = 0.036). (**C**) Calprotectin serum concentrations are significantly higher in critically ill patients at day 7 following ICU admission compared to the respective concentrations at day 1. (**D**) Patients with calprotectin serum concentrations below the 50th percentile (4.188 µg/mL) at day 7 following ICU admission do not show significant differences regarding overall survival compared to patients above the 50th percentile (*p* = 0.417). (**E**) ICU patients with increasing calprotectin concentrations between day 1 and 7 have a significantly better OS compared to patients with decreasing calprotectin concentrations. (**F**) Septic patients with increasing calprotectin concentrations between day 1 and 7 have a significantly better OS compared to patients with decreasing calprotectin concentrations. * *p* < 0.05.

Based on these results, we assumed that higher calprotectin concentrations might affect overall survival (OS) in septic patients. Therefore, we compared the OS in the subgroup of septic patients with high or low calprotectin serum concentrations. Using the median calprotectin serum level (2.763 µg/mL) as cut-off value, Kaplan–Meier curve analysis revealed a trend towards an impaired OS in septic patients with calprotectin serum concentrations at ICU admission (*p* = 0.057, Figure 3A). We subsequently established an ideal prognostic cut-off value (see Materials and Methods for details). When applying this ideal cut-off value, septic patients with a baseline calprotectin serum level above 2.001 µg/mL had a significantly impaired OS compared to patients with calprotectin concentrations below this cut-off value (*p* = 0.036, Figure 3B). The median OS in the calprotectin-low group was 342 days but was not reached in the calprotectin-high group.

To further underline the prognostic value of circulating calprotectin, we next performed univariate Cox-regression analysis. Here, a calprotectin serum concentration above the ideal cut-off value at the first day of ICU admission turned out as a significant predictive factor for overall mortality (HR: 4.002, 95% CI: 1.190-13.459, *p* = 0.025). Bootstrapping analysis for internal validation was complemented, which also revealed a significant result (*p* = 0.025). To exclude overestimation of effects, Cox regression was repeated including calprotectin baseline concentrations as a continuous variable; here, calprotectin concentrations at day 1 of ICU admission did not significantly predict overall mortality but showed a trend (*p* = 0.154).

For a subset of critically patients (*n* = 67), serum samples at day 7 following ICU admission were available. Interestingly, calprotectin serum concentrations were significantly higher at day 7 when compared to the respective values at ICU admission (*p* = 0.030, Figure 3C). To evaluate whether the prognostic role of circulating calprotectin also existed during the course of ICU treatment, we again compared the overall survival between patients with high or low calprotectin values at day 7. Applying the 50th percentile of calprotectin serum concentrations at day 7 (4.188 µg/mL, Figure 3D), no difference regarding the overall survival could be shown for critically ill patients above compared to patients below this cut-off. 

Finally, we analyzed if the individual course of calprotectin serum concentrations might have an impact on the critically ill patients’ OS. Therefore, we calculated the delta of calprotectin serum concentrations between day 7 and admission (day 1). Here, patients with an increase in calprotectin concentrations between day 1 and day 7 of ICU admission had a significantly improved overall survival compared to patients who showed decreasing calprotectin concentrations during the first week of critical illness (*p* = 0.009, Figure 3E). Correspondingly, septic patients with increasing values of calprotectin serum concentrations within the first week of ICU admission displayed a significantly better overall survival (*p* = 0.033, Figure 3F). Both in the study group of all patients as well as in the subgroup of septic patients, the categorized variable of increasing calprotectin serum concentrations (delta between day 1 and day 7 of >1) remained a significant predictor for reduced overall mortality according to the univariate Cox regression analysis (HR 0.304; 95% CI 0.117–0.789, *p* = 0.014 for all critically ill patients; HR 0.335; 95% CI 0.116–0.968, *p* = 0.043). Statistical significance was also reached for the calprotectin delta as a continuous variable (HR 0.850; 95% CI: 0.761–0.948, *p* = 0.004 for all patients; HR 0.861; 95% CI: 0.761–0.974, *p* = 0.018 for septic patients). Both results were confirmed by bootstrapping as an internal validation (*p* = 0.003 for all patients and *p* = 0.010 for septic patients).

Next, we aimed at investigation whether dynamics of calprotectin serum concentrations represent an independent predictor of critically ill patients’ outcome. We therefore evaluated a wide range of clinicopathological parameters (age, sex, BMI) as well as various laboratory parameters of organ dysfunction including markers of systemic inflammation (CRP, PCT) and an impaired liver (bilirubin) or renal (creatinine) function in a univariate Cox-regression analysis. In multivariate Cox-regression analysis with a stepwise backward selection of variables procedure including parameters with a potential prognostic relevance in univariate testing (*p* < 0.200), the delta of calprotectin serum concentrations remained an independent prognostic marker for OS (HR: 0.815, 95% CI: 0.720–0.922, *p* = 0.001; Table 3).

## 4. Discussion

In this study, we demonstrate that serum concentrations of calprotectin are increased in critically ill patients as well as septic patients at ICU admission compared to controls. High calprotectin concentrations were indicative for an unfavorable clinical outcome since septic patients with high baseline calprotectin concentrations had a more severe clinical course and displayed an impaired overall survival. Importantly, changes in calprotectin serum concentrations during the first week of admission were prognostically relevant, since patients with increasing calprotectin concentrations were observed to have an improved overall survival. These results are in line with previous data demonstrating serum-based calprotectin to be increased in critically ill patients [19,20,21,22].

The patients submitted to our ICU showed a broad spectrum of specific diseases and comorbidities that might influence systemic calprotectin concentrations as well as overall survival. For instance, 10% of the included patients suffered from hematological malignancy or solid tumor. Moreover, calprotectin concentrations were significantly lower in patients with CAD compared to healthy controls. However, one would have expected increased calprotectin serum concentrations in CAD patients as CAD is referred to as a chronic inflammatory disease and patients with myocardial infarction show increased calprotectin concentrations [23]. We argue here, that due to a selection bias, CAD patients are underrepresented in our cohort of critically ill patients. Therefore, we do not claim to adequately characterize calprotectin serum concentrations in critically ill patients. However, our data point at further triggers of increased calprotectin serum levels in critically ill patients other than an underlying cardiovascular disease.

Moreover, and also due to selection bias, only 7 patients included in our analysis had a previous diagnosis of liver cirrhosis. These patients had significantly lower calprotectin concentrations compared to non-affected patients. Previous data revealed differential calprotectin serum concentrations in patients with different etiologies of liver cirrhosis and it was discussed that elevated calprotectin concentrations indicate bacterial infections in cirrhotic patients [24]. One possible explanation of lower calprotectin concentrations in our included cirrhosis patients compared to non-cirrhotic patients next to a selection bias is that only 3 out of 7 (43%) fulfilled the diagnostic criteria of sepsis compared to a prevalence of sepsis of 68% in the non-cirrhotic cohort. 

We describe significantly lower calprotectin serum concentrations in COPD compared to non-COPD patients. However, calprotectin concentrations in the included COPD patients were still increased compared to serum concentrations in patients with exacerbated COPD described in the literature (median of calprotectin serum concentrations in COPD patients included in our cohort 2079.7 ng/mL compared to a median of 176 ng/mL in exacerbated COPD [25]) pointing at further triggers of circulating calprotectin expression in the included critically ill patients of our cohort with coincidental COPD.

In both critically ill as well as septic patients, calprotectin concentrations correlated with serum concentrations of CRP but not leukocyte count or procalcitonin serum concentration. Calprotectin expression is linked to systemic inflammation as calprotectin is derived from neutrophils and macrophages participating in inflammatory processes. Molecular pathways resulting in upregulated calprotectin concentrations have been established in different models of bacterial infections [7,8,26]. Here, the protein complex of S100A8 and S100A9 magnifies the inflammatory response by accelerating the cytokine release of immunocytes and neutrophil recruitment [26]. Our data describing calprotectin as an early biomarker of bacterial infections in critically ill patients are in line with previous studies [20]. However, calprotectin did not indicate the site of infection, since patients with different septic foci did not show significantly different calprotectin serum concentrations. 

Correlation analyses furthermore revealed a trend towards higher creatinine concentrations and a significant correlation with duration of RRT in critically ill patients with higher calprotectin concentrations. These data complete previous studies describing higher calprotectin serum concentrations in critically ill patients fulfilling the criteria of acute kidney injury [27]. Calprotectin as a potential biomarker in sepsis-related organ failure is furthermore supported by the strong correlation of calprotectin concentrations with mechanical ventilation parameters: In both critically ill as well as septic patients with respiratory failure, calprotectin indicated respiratory deterioration. In line with that, calprotectin has previously been shown to be elevated in pulmonary fluid of patients suffering from lung injury and to contribute to amplification of ventilator-induced lung injury in mice [28,29]. Interestingly, recent investigations on COVID-19 identified rising calprotectin serum concentrations in COVID-19 patients requiring higher oxygen fractions and furthermore discovered elevated calprotectin concentrations to distinguish severe from mild COVID-19 [30]. Exogenous application of the S100A8/A9 complex has resulted in further amplification of pulmonary inflammation in murine models of lung injury [29]. Our human and recent experimental data should encourage further studies to investigate potential therapeutic effects of calprotectin extraction or neutralization in respiratory failure in critically ill patients.

Larsson et al., among others, revealed a predictive ability of calprotectin serum concentrations regarding short-term mortality in ICU patients with sepsis [17,31]. Our study is the first to investigate the prognostic value of calprotectin serum concentrations in long-term and overall survival (OS) of critically ill patients with and without sepsis. We show that non-survivors after 180 and 365 days show a trend towards higher calprotectin concentrations. Especially, septic patients who died within 180 and 365 days after ICU admission have significantly higher calprotectin concentrations at ICU admission. These results are further reflected by analysis of OS of septic patients: At the respective optimal cut-off value that was established using a recently described biometric software [18], high calprotectin concentrations (>2.001 μg/mL) turned out as a powerful predictor of OS in septic patients and its prognostic impact was furthermore confirmed by bootstrapping as an internal validation. Our data therefore appropriately follow up on decreased short-term survival in patients with increased calprotectin concentrations. However, when we included calprotectin serum concentrations at day 1 as a continuous parameter to avoid overestimation of effects, Cox regression analysis only revealed a trend towards an impaired overall survival. Nevertheless, our data demonstrate, that increased calprotectin serum concentrations reflect severity of systemic inflammation in critically ill patients as shown for other acute as well as chronic inflammatory diseases. Experimental approaches blocking the S100A8/A9 activity exerted beneficial effects on disease activity in autoimmune and inflammatory bowel disease [32].

Interestingly, both critically ill patients as well as septic patients who had increasing calprotectin concentrations between day 1 and day 7 of ICU admission showed favorable outcome as described by improved overall survival. We therefore argue that excessive calprotectin concentrations during the first phase of systemic inflammation reflects a severe inflammatory response accompanied by impaired long-term survival. However, calprotectin appears to display a double-edged function in critical illness as well as sepsis, as based on our data after the acute phase of critical disease, ICU patients could benefit from a further increase of calprotectin secretion during systemic inflammation. In this context, calprotectin has been described in previous studies to regulate inflammatory responses, thereby preventing hyper-inflammation [33]. Beyond that, calprotectin was identified to exert anti-inflammatory, antimicrobial, e.g., beneficial effects during latter stages of inflammation as it was shown to modulate production of pro-inflammatory mediators and also trap pro-inflammatory cytokines in vitro [34,35]. It was moreover even described to exert bacteriostatic activity [36]. S100A8/A9 binds and controls the concentrations of metals such as Zn^2+^ and Mn^2+^ required for bacterial growth [37] and enhances the antimicrobial efficiency of neutrophil phagocytosis, thereby accelerating clearance of pathogens [38,39]. In line with that, in a murine model of *Staphylococcus aureus* infection, S100A8/A9 deficient mice showed progression of pneumonia in contrast to wild type mice [40]. These experimental studies, supported by our human data, should encourage further exploration of potential therapeutic effects of calprotectin in systemic inflammatory response syndromes, as well as in sepsis.

There are some limitations of our study. First, all data analyses were performed in an exploratory single centre cohort of adult patients leading to a potential lack of generalizability of results. This includes that our data are not comparable to further studies on calprotectin in pediatric critical illness patients, since patients below 18 years were not included in our study. Secondly, we included critically ill patients with a broad spectrum of underlying disease etiology. We assume that this approach provides a certain amount of transferability of results. However, it might result in disease-specific confounders. Furthermore, our study lacks a validation cohort. This limitation could partly be compensated by additional bootstrapping analysis for internal validation. Although we were able to evaluate calprotectin serum concentrations in a small subset of patients at day 7 following ICU admission in addition to day 1, our study lacks calprotectin measurements at further time-points during ICU treatment. Moreover, participants with missing data were omitted from the analysis, resulting in a data analysis bias according to the PROBAST tool [41]. Therefore, we argue, that the observed prognostic impact of dynamics in calprotectin concentrations needs to be validated in larger, multi-centre cohorts of ICU patients to fully understand the role of calprotectin in predicting patients’ outcome.

In summary, serum calprotectin concentrations are significantly increased in critically ill patients with sepsis and associated with renal and respiratory dysfunction. High calprotectin concentrations at ICU admission predict long-term mortality risk. Strikingly, our study is the first to demonstrate a prognostic value of rising calprotectin concentrations during ICU treatment. 

## Figures and Tables

**Table 1:** Baseline patient characteristics at the time point of admission and calprotectin serum concentrations at day 1 and day 7.

Parameter	Patients
Number	165
Gender	
Female (%)	40.6
Male (%)	59.4
Age, median in years (range)	64 (18–90)
BMI, median (range)	25.8 (15.9–86.5)
Diabetes mellitus type 2 (%)	31.6
Coronary artery disease (%)	22.3
COPD (%)	29.8
Malignant disease (%)	10
Solid tumour	5
Hematological malignancy	4
Main diagnosis/reason for admission (%)	
Sepsis	65.5
Infectious focus of sepsis (%)	
Pulmonary	55.6
Abdominal	19.4
Urinary tract	2.8
other	22.2
Liver cirrhosis	4.2
Cardiopulmonary disease	13.9
Acute liver failure	1.2
Acute pancreatitis	5.5
Gastrointestinal bleeding	2.4
Other	7.3
APACHE-II score at day 1	17 (3–43)
<17 (%)	50.7
>17 (%)	49.3
SOFA score at day 1	9 (0–17)
<9 (%)	56.6
>9 (%)	43.4
Mechanical ventilation demand (%) at day 1	41.8
Vasopressor demand (%) at day 1	62.4
Death on ICU (%)	19.4
30 d mortality (%)	23.7
90 d mortality (%)	32.3
180 d mortality (%)	40.4
365 d mortality (%)	57.0
long-term mortality (%)	40.0
Calprotectin [µg/mL]	
Day 1	2.482 (0.004–12.5)
Day 7	4.073 (0.544–3.023)

For quantitative variables, median and range (in parenthesis) are given. Abbreviations are: BMI, body mass index; COPD, chronic obstructive pulmonary disease; SOFA, sequential organ failure assessment; APACHE, acute physiology and chronic health evaluation; ICU, intensive care unit.

**Table 2:** Linear regression analysis of calprotectin with baseline characteristics, markers of inflammation, markers of organ dysfunction, other laboratory markers, and clinical scores at day 1 of ICU admission.

	All Patients				Sepsis	
	r	95% CI	*p*	r	95% CI	*p*
**Baseline Characteristics**						
Age	−28.25	−68.75–12.25	0.170	−40.14	−91.61–11.33	0.125
BMI	51.24	−8.66–111.14	0.093	40.29	−25.54–106.13	0.227
ICU Days	4.95	−29.50–39.39	0.777	−22.81	−62.51–16.88	0.257
Hospital days	15.180	−12.25–42.61	0.275	−2.30	−35.45–30.86	0.890
**Markers of Inflammation**						
Leukocytes	39.37	−17.32–96.06	0.172	25.53	−39.0–90.03	0.434
CRP	17.63	10.64–24.62	<0.001 ***	17.67	7.01–28.33	0.001 **
Procalcitonin	18.38	−4.0–40.73	0.106	15.16	−9.35–39.67	0.221
IL-6	−0.12	−0.37–0.13	0.340	−0.151	−0.413–0.11	0.255
**Markers of Organ Dysfunction**						
Creatinine	287.92	−7.14–582.98	0.056	221.73	−129.37–572.84	0.213
GFR	−27.21	−66.69–12.27	0.175	−20.08	−69.79–29.62	0.423
Duration of RRT in Days	186.97	59.41–314.53	0.004 **	130.45	−10.99–272.0	0.070
Urea	12.74	1.22–24.26	0.030 *	10.79	−4.09–25.66	0.153
Sodium	−43.85	−148.60–60.91	0.409	−38.57	−163.89–86.76	0.543
Kalium	−560.22	−1465.68–345.23	0.223	−556.72	−1764.11–650.67	0.362
AST	−0.190	−1.02–0.64	0.650	0.045	−2.52–2.62	0.972
Bilirubin Total	−26.53	−335.50–282.44	0.865	579.00	−116.06–1274.06	0.101
LDH	0.90	−0.37–2.17	0.165	3.77	−0.171–7.72	0.061
Lactate	114.17	−158.44–386.78	0.409	185.17	−180.55–550.89	0.317
NT-proBNP	0.032	−0.06–0.13	0.503	0.024	−0.09–0.13	0.671
**Clinical Scores**						
APACHE II	−25.23	−110.75–60.29	0.560	−89.70	−202.80–23.40	0.118
SOFA	−30.19	−229.89–169.51	0.764	−151.36	−481.0–162.07	0.323
SAPS2	−43.06	−124.12–38.00	0.291	−18.56	−119.84–82.73	0.711
**Mechanical Ventilation Parameters**						
FiO_2_ at Day 1	64.23	33.13–95.33	<0.001 ***	52.61	10.0–95.22	0.017 *
P_max_ at Day 1	106.58	23.98–189.19	0.012 *	114.37	15.37–213.37	0.025*
PEEP at Day 1	315.12	145.40–484.83	<0.001 ***	358.23	159.59–556.86	0.001 **
Duration of Mechanical Ventilation in Days	−0.23	−1.95–1.50	0.796	−1.38	−3.33–0.58	0.166

Linear regression analysis was performed to test significance; the regression coefficient is depicted as “r” with 95% confidence interval (CI) with * *p* < 0.05; ** *p* < 0.005, *** *p* < 0.001. Abbreviations: BMI, body mass index; CRP, C-reactive protein; IL-6, interleukin 6; GFR, glomerular filtration rate; RRT, renal replacement therapy; AST, aspartate aminotransferase; LDH, lactate dehydrogenase; BNP, brain natriuretic peptide; APACHE, acute physiology and chronic health evaluation score; SOFA, sepsis-related organ failure assessment score; SAPS2, simplified acute physiology score; FiO_2_, inspired oxygen fraction; P_max_, maximum ventilation pressure; PEEP, positive endexpiratory pressure.

**Table 3:** Univariate and multivariate Cox regression analysis for the delta of calprotectin serum concentrations between day 1 and day 7 in critically ill patients.

Parameter	Univariate Cox Regression		Multivariate Stepwise Backward Cox Regression	
	*p*-Value	Hazard Ratio (95% CI)	*p*-Value	Hazard Ratio (95% CI)
Calprotectin delta ^#^	0.004 **	0.850 (0.761–0.948)	0.001 **	0.815 (0.720–0.922)
Age	0.002 **	1.041 (1.014–1.068)	0.001 **	1.043 (1.018–1.070)
Sex	0.518	0.811 (0.429–1.532)		
BMI	0.119	0.955 (0.901–1.012)	0.016 *	0.921 (0.860–0.985)
CRP	0.113	1.003 (0.999–1.007)	0.140	1.003 (0.999–1.007)
Procalcitonin	0.851	1.001 (0.990–1.012)		
Creatinine	0.498	1.039 (0.931–1.159)		
Bilirubin	0.339	1.167 (0.850–1.601)		
Lactate	0.295	1.081 (0.935–1.249)		

Multivariate Cox regression was performed applying a stepwise backward variable selection procedure. ^#^ Calprotectin delta was calculated by subtraction of calprotectin serum concentrations at day 1 of ICU admission from serum concentrations at day 7 of ICU admission. * *p* < 0.05; ** *p* < 0.005.. Abbreviations: BMI, body mass index; CRP, C-reactive protein.

## Abbreviations

ICU	medical intensive care unit
APACHE	acute physiology and chronic health evaluation score
SOFA	sequential organ failure assessment score
SAPS2	simplified acute physiology score
BMI	body mass index
CAD	coronary artery disease
COPD	chronic obstructive pulmonary disease
CRP	C-reactive protein
IL-6	interleukin 6
RRT	renal replacement therapy
GFR	glomerular filtration rate
AST	aspartate aminotransferase
BNP	brain natriuretic peptide
LDH	lactate dehydrogenase
FiO_2_	inspiratory oxygen fraction
P_max_	maximum airway pressure
PEEP	positive end-expiratory pressure
OS	overall survival
